# Image Acquisition Device for Smart-City Access Control Applications Based on Iris Recognition

**DOI:** 10.3390/s21186185

**Published:** 2021-09-15

**Authors:** Damjan Zadnik, Andrej Žemva

**Affiliations:** 1 Iretec d.o.o., 4000 Kranj, Slovenia; 2 Faculty of Electrical Engineering, University of Ljubljana, 1000 Ljubljana, Slovenia; andrej.zemva@fe.uni-lj.si

**Keywords:** iris recognition, image acquisition, access control, image sensor, image processing

## Abstract

In this work, we present an eye-image acquisition device that can be used as an image acquisition front-end application in compact, low-cost, and easy-to-integrate products for smart-city access control applications, based on iris recognition. We present the advantages and disadvantages of iris recognition compared to fingerprint- or face recognition. We also present the main drawbacks of the existing commercial solutions and propose a concept device design for door-mounted access control systems based on iris recognition technology. Our eye-image acquisition device was built around a low-cost camera module. An integrated infrared distance measurement was used for active image focusing. FPGA image processing was used for raw-RGB to grayscale demosaicing and passive image focusing. The integrated visible light illumination meets the IEC62471 photobiological safety standard. According to our results, we present the operation of the distance-measurement subsystem, the operation of the image-focusing subsystem, examples of acquired images of an artificial toy eye under different illumination conditions, and the calculation of illumination exposure hazards. We managed to acquire a sharp image of an artificial toy eye sized 22 mm in diameter from an approximate distance of 10 cm, with 400 pixels over the iris diameter, an average acquisition time of 1 s, and illumination below hazardous exposure levels.

## 1. Introduction

Computer vision technology uses digital image processing to extract information from images, which is then used in decision algorithms employed by many applications. State-of-the-art, multi-vision systems represent an extension of monocular vision systems, where a wider range of 3D geometric information is obtained [1]. For example, in a recent object detection application, Tang et al. [2] used a dynamic real-time, mark-free, four-ocular stereoscopic visual tracking system to measure the surface deformation of large-field recycled concrete-filled steel tube columns. Computer vision technology can also be used to automate biometrics processes that are used for the identification and authentication of individuals by measuring and analyzing their personal traits: fingerprints, iris, hand geometry, voice, face, vascular pattern, palm-print, or behavioral characteristics (e.g., signature, typing pattern, and gait). Automated biometric technology provides an advanced methodology with an advantage over traditional access control methods. Pin codes or passwords can be forgotten, and identification cards or keys may be lost or stolen. Biometric traits are difficult to steal or forget. Owing to its unique characteristics and high security, biometric technology is used in a variety of applications [3]. In a verification application scenario, an acquired biometric sample is compared against previously stored samples for matching. In an identification scenario, a biometric sample is acquired with the task of identifying the unknown sample as matching a previously acquired known sample. In both scenarios, four possible matching outcomes are possible: true accept, false accept, true reject, or false reject [4].

The overall global biometrics market is expected to grow rapidly from USD 19.5 billion in 2020 to USD 44.1 billion by 2026, registering a compound annual growth rate (CAGR) of 14.8% [5]. The global market for contactless biometrics will generate more than USD 9 billion in revenue during 2021, and it is expected to grow at a robust 16% CAGR over the next decade [6]. The demand for contact-based biometric systems has been affected by the COVID-19 pandemic. The demand for such contact-based biometric systems is likely to fall drastically to avoid the spread of coronavirus. Similarly, contactless biometric systems, such as face recognition, iris recognition, and voice recognition, are expected to witness a boost in demand post-COVID-19 [7].

### 1.1. Iris, Fingerprint, and Face Recognition

**Figure 1:**
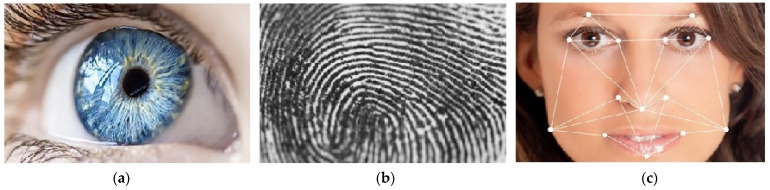
Diversity and accuracy of biometrics systems: (a) iris texture—high diversity and accuracy; (b) fingerprint pattern—middle diversity and accuracy; (c) face image—low diversity and accuracy.

Iris, fingerprint, and face recognition are the most well-known forms of biometric security. As shown in Figure 1 [8], when comparing iris textures to fingerprint patterns or to face images, iris textures have the highest diversity. This feature makes iris recognition the most accurate and, therefore, also the most reliable non-invasive biometric system. However, what are the other pros and cons of these three systems?

Iris recognition is a method of biometric authentication that uses pattern recognition techniques based on high-resolution images of the irises of an individual’s eye. The iris is formed in the early stages of life, and once it is fully formed, its texture remains stable throughout a person’s life. Since the iris is an internal organ, it is well protected against damage and wear. Iris recognition is an excellent security technique, especially if it is performed using infrared illumination to reduce specular reflection from the convex cornea. Its efficiency is rarely impeded by the presence of glasses or contact lenses. However, it is difficult to perform iris recognition from a distance further than a few meters. A frequently encountered problem when the technology is introduced is resistance from users. People usually undergo an unpleasant experience when having their eyes scanned. They must also adopt a certain position, which can cause discomfort. Iris scanners are also relatively expensive compared to other recognition modalities [8,9].

Fingerprint recognition is the oldest and most widely used recognition modality. Most known examples of its application include mobile phones or laptops, building access, or car doors. Fingerprints are typically composed of ridges and furrows, and their uniqueness is determined by the patterns made by the ridges, minutiae points, and furrows, which remain unchanged throughout the life of an individual. An identification system based on fingerprint recognition looks for specific characteristics in the pattern. However, fingerprints may be damaged or even worn away and, consequently, cannot be recognized or recorded. On the other hand, fingerprint identification is already familiar to the public and is widely accepted. It can be easily integrated into existing applications. Users can easily learn to use this system, as it is intuitive and needs no special training. The advancements of technology have led to small and inexpensive fingerprint readers, and the deployment of these systems has increased in a wide range of applications [8,9].

Face recognition has developed rapidly in recent years and is an excellent candidate if a system is needed for remote recognition. Although it may not have high accuracy, one primary advantage of this system is that it does not need the cooperation of the person under identification. Face recognition may work even when the subject is unaware of being scanned. A face recognition system analyzes the shape and position of different parts of the face to determine a match. It is a hands-free and non-intrusive identification method that can be utilized in static or dynamic applications. However, there are several factors that can affect the accuracy of facial biometrics: it might not work well under poor lighting conditions; the face of a person changes over time; the shape of the face is also affected by variations in facial expressions [8,9].

### 1.2. Iris Recognition Standards

**Figure 2:**
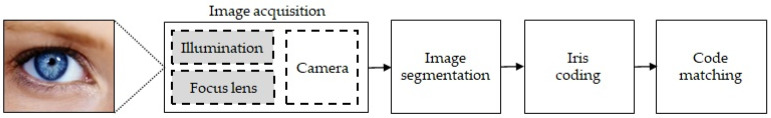
Basic principles of an iris recognition system.

The most important work in the early history of iris biometrics is that of Daugman [4]. In 1993, Daugman [10] published the first academic paper proposing an actual method for iris recognition, just before his corresponding US patent [11] was also issued. Today, Daugman’s IrisCode algorithm is implemented in most systems deployed worldwide, which operate as licensed executables [12]. The basic principle of an iris recognition system is shown in Figure 2. First, a sharp image of the eye is acquired. Then, the acquired image is segmented, and the iris image is extracted. In the iris coding phase, some mathematical transformation is applied on the extracted iris segment. In the code-matching phase, the results of this transformation are used in search algorithms for the best-matching candidate in a database of stored iris codes [13].

A well-structured and widely implemented standard can create entirely new markets and promote competition. The ISO/IEC19794-x family of standards for biometric data interchange formats consists of 14 parts. These standards are needed for applications where biometric data, which are stored in a standard format, must be processed using compatible equipment. The most widely implemented standard from this family is ISO/IEC19794-5, which specifies the format requirements for the storage of facial images. It is used by national passport issuers and passport readers on immigration checkpoints. Iris recognition interoperability requires that data records are both syntactically and semantically understood by the receiving system. Iris recognition standardization efforts began in 2002, after the September 11 attack. At that time, the only commercial entity in the field was Iridian, which held Daugman’s patent rights. Iridian volunteered the first standard draft; in 2005, this led to the almost identical ISO/IEC19794-6 standard [14]. The standard specifies the image data format, image properties, image quality, and image capture recommendations. For example, the standard specifies an image size of 640 × 480 pixels and an acquired iris diameter of 200 pixels.

Exposure to optical radiation has been linked with several reactions that fall within the category of photobiological effects and have been shown to be of risk to the skin and eye. The IEC62471 standard is recognized in many countries as the key standard addressing photobiological safety issues. According to this standard, individuals in the vicinity of lamp systems must not be exposed to levels exceeding the exposure limits, which represent the conditions to which it is believed that nearly all individuals in the general population may be repeatedly exposed without adverse health effects [15].

### 1.3. Commercial Solutions for Smart-City Access Control Applications and the Market Opportunity

**Figure 3:**
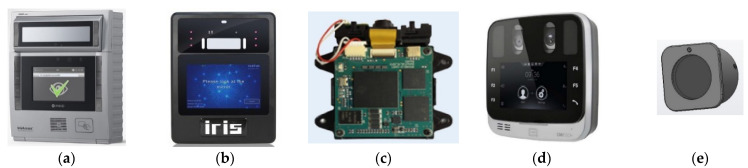
Examples of existing access control devices, based on iris recognition, intended for smart-city applications: (**a**) IRISID-iCAM7; (**b**) GRANDING-IR7pro; (**c**) IRITECH-MO2121; (**d**) THOMAS-TA-EF-45; (**e**) proposed concept design.

To access our smart-city homes using iris recognition technology, a compact, low-cost, and easy-to-integrate solution is required. Several commercial solutions using access-control devices based on iris recognition, intended for smart-city applications, exist on the market today. Figure 3a–d shows four examples of such solutions, and Table 1 lists their typical technical characteristics [16,17,18,19]. The main drawback of existing commercial solutions is that they are designed to be mounted on a wall, near the entrance door. Their price, the cost of installation excluded, is in the range of USD 1000. They are not designed for simple push-in-door installation, which would simplify the integration and maintenance for door manufacturers. Fingerprint readers are also important competitors. They are widely adopted, small, inexpensive, and designed to be integrated into the door or even within the doorknob. Therefore, an affordable solution that can replace fingerprint readers is required. Figure 3e shows our proposed solution, which is a design concept. The last row in Table 1 shows its basic technical characteristics. Our design will be compact and intended for direct door-mounting. The device will of course conform to illumination exposure limits. It will use a local, non-standardized database of 30 users, which will be based on images that are 800 × 600 in size. Since we use an off-the-shelf, low-cost color camera, visible illumination will be used. The capture distance will be from 7 to 13 cm. The target access time will be less than 2 s, which is comparable to fingerprint readers. The target production cost should be less than USD 200 at the production level of 5000 units per year in the first production year.

### 1.4. Purpose, Goal, and Innovation of This Work

The arguments discussed in Section 1.3 led us to the purpose of this work, which was to verify if a compact access control device, based on iris recognition technology and intended for easy integration into smart-city doors, can be built at a reasonable cost.

Our project is divided into two stages. In this work, we present the results of the first stage. The main goal of this work was to develop a device that performs FPGA-based real-time image acquisition of a sharp grayscale image of an illuminated eye, using a camera module that integrates a Bayer-based color image sensor and a motorized lens. In the second stage, the device will be further miniaturized, and other iris recognition phases (iris segmentation, iris coding, code matching) will be added as the software upgrades.

The main innovation of this work is the method for real-time processing of the raw RGB image frames acquired from the camera module. In this work, we present a combined real-time image-processing technique that performs two image-processing tasks that are executed in real-time and in parallel on an FPGA. The first task is image conversion from raw RGB to grayscale. The second task is image-focusing, which is performed on raw RGB images. To the best of our knowledge, the described image-processing method has not yet been presented in any iris image acquisition or iris recognition applications.

### 1.5. Structure of This Work

This paper is organized as follows. In Section 2, the scientific background and the related research work are presented. This chapter is divided into four subsections: iris illumination and acquisition, iris image quality metrics and image focusing, image sensor demosaicing, and image conversion from color to grayscale. Section 3 presents the research method and materials used. In Section 4, the results of the research are presented and discussed. Conclusions and future work are presented in Section 5.

## 2. Scientific Background and Related Research Work

### 2.1. Iris Illumination and Acquisition

Eye color does not vary smoothly across the iris. Instead, it is arranged in clusters of similar colors that can have abrupt boundaries arising from structures within the iris. The perception of iris color, based on light reflection, light scattering, light diffraction, and light absorption on the iris, is a complex phenomenon, combining the effects of pigmentation, texture, fibrous tissue, and blood vessels in the iris. Shorter wavelengths of light are commonly reflected or scattered, while the longer wavelengths are typically absorbed by the iris. The amount of texture information, extractable from a given iris, is dependent on the wavelength under which it is illuminated. Traditionally, only a narrow band of the near-infrared (NIR) spectrum (700–900 nm) is utilized for iris recognition, since this alleviates any physical discomfort from illumination, reduces specular reflections, and increases the amount of texture captured for certain iris colors. The diffuse reflectivity of an iris is approximately 10% in the NIR band. High NIR illumination levels may be hazardous because the eye does not instinctively respond with its natural protective mechanisms, such as aversion, blinking, and pupil contraction. On the other hand, low visible light illumination levels, in unconstrained imaging setups, can degrade the quality of captured data [20,21,22].

The constraints in all commercial iris recognition systems are mostly driven by the image acquisition process, which imposes the subject’s position and motion during the recognition process. The metrics for the required degree of cooperation are the capture volume, the standoff distance, and the acquisition time. At any distance from which they are taken, the iris images must provide sufficient information for the task of iris recognition. An iris-imaging system captures a single or dual iris image at a given distance using illumination that is over a prescribed illumination wavelength band. Iris cameras that operate at approximately 30 cm or less from the subject typically resolve 200 pixels across the iris diameter. All commercial iris imaging systems conform to eye-safety standards that limit the irradiance and radiance values [23,24].

Matey et al. [23] presented the results of the “Iris on the Move” project. The developed system was able to capture iris images of sufficient quality for iris recognition, while the subject was moving at a normal walking speed, through a confining portal. The acquisition camera was approximately 3 m in distance, the image capture volume was 0.2 m × 0.4 m × 0.1 m, and a synchronized NIR LED illumination was used. Images with approximately 100 pixels across the iris were obtained. Most subjects were able to wear eyeglasses or contact lenses. The overall recognition rate for all subjects was 78%. Yoon et al. [25] proposed the usage of a pan-tilt-zoom camera at a distance. The system covered a capture volume of 1 m × 1 m × 1 m, at a stand-off distance, and ranged between 1.5 m and 2.5 m. Two NIR illuminators were used and a stepping motor was used to control the focus. To acquire an iris, the face was first detected, using the Viola-Jones algorithm. Then, the pan-tilt system was used to place the face in the center of the image. Lastly, the iris was detected by the circular edge-detector algorithm. Dong et al. [26] presented an iris image acquisition system that was self-adaptive to users. Two low-resolution cameras were used, one for face acquisition and the other for iris acquisition. Once the face region was detected by the face camera, the iris was captured by the iris camera. The subject was not moving, and the capture volume was 0.6 m × 0.4 m × 0.4 m. The average capture time was 5.1 s. He et al. [27] designed an iris camera with the goal of being more economical than commercial alternatives while still acquiring high-quality images. They used a 480 K pixel image sensor with a custom lens and a fixed focus at 25 cm, also using an 880 nm NIR LED illumination and an NIR-pass filter to minimize specular reflections on the iris. Hu and Si [28] presented an image acquisition and real-time detection system using a convolutional neural network. Image acquisition and processing were achieved with an Altera Cyclone IV FPGA, a DDR memory, and a digital image sensor OV7725. Image resolution was set to 640 × 480 pixels, and the RGB565 image output data format was used. The system was connected to a PC through a USB interface. The NIR illumination and the manually adjustable focus lens were used to collect iris images at distances of 1.5 m, 1.6 m, and 1.7 m.

### 2.2. Iris Image Quality Metrics and Image Focusing

Iris sample quality has several applications. It can be used at different processing levels in iris recognition systems, for example, at the image acquisition stage, at the image enhancement stage, or at the matching stage. Iris image quality is evaluated by means of quality metrics to quantify what information an iris image contains, or if the image should be discarded or further processed. The metrics can be broadly divided into two groups. The first group of metrics includes environmental and camera effects. The second group of metrics belongs to the unconstrained presentation of a subject, for example, insufficient illumination, defocus blur, off-angle presentation, and occlusion [29]. The image quality metric can improve a system’s performance. However, there is no generally accepted measure of overall iris image quality [4]. Most authors from the research field of iris image acquisition use the image focus metric for assessing motion or defocus blur. Active auto-focusing (AF) or passive AF may be used to capture a sharp image in digital cameras. While the active AF utilizes a distance sensor to measure the distance between the lens and the object to adjust the focus lens position, the passive AF calculates the image sharpness value using pixel processing [30,31]. Different methods for image sharpness evaluation exist in the literature. In evaluations based on gradient, focused images have larger gray or color-change levels than those that are defocused. In evaluations based on correlation, blurred images have a stronger pixel correlation, whereas, in evaluations based on statistics, the clearest image contains the maximum amount of entropy. Transform-based evaluations use Fourier or other transforms to evaluate the frequency spectrum of an image. Focused images contain more high-frequency components because they obtain sharper and clearer edges [32].

Daugman’s system assessed the focus of the image in real time by looking at the power in the middle and upper bands of the 2D Fourier spectrum. He used an 8 × 8 convolution kernel to calculate the Fourier transform [4,10]. He et al. [33] developed a clear iris image acquisition system with a fixed focus lens and best focus distance of 30 cm. An infrared sensor was used for distance measurement. They then used the 2D Fourier transform of the image to evaluate the power of the high-frequency components in the image. Kang and Park [34] proposed a 5 × 5 convolution kernel, similar to Daugman’s kernel. To test the focus assessment method, they artificially generated blurred iris images of the Casia 1.0 iris image database, using a Gaussian kernel mask with various σ values. Yousefi et al. [35] evaluated the following gradient-based sharpness functions: Vollath’s F4, Vollath’s F5, the variance of the image, squared gradient, sum of differences across rows, sum of differences across rows and columns, and the sum of Laplacians, in terms of accuracy and computation time on a PC platform. The accuracy was measured as a percentage of matching the true focus position. The sum of differences method gave the fastest computational time, with a 92% accuracy, while the square gradient method was more than three times slower, with a 77% accuracy. Yang and Wang [36] proposed an iris image quality evaluation method based on a gray-level evaluation, where iris image quality was reflected by evaluating a weighted average of three quality metrics: iris position, iris visibility, and iris clarity. The proposed quality index was tested on the images from the Casia 1.0 iris image database, where they correctly detected 95.7% of irises.

### 2.3. Image Sensor Demosaicing

**Figure 4:**
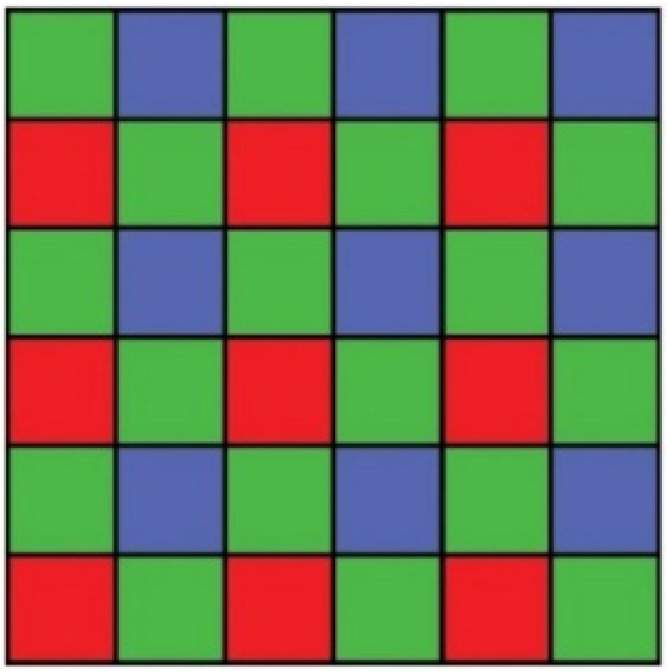
Bayer color filter array (CFA) pattern, as widely used in color CMOS sensors [38].

Each pixel in a digital color image is composed of red, green, and blue (RGB) color channels. A color digital camera would require three image sensors, one for each color channel. To reduce costs, digital camera manufacturers employ a single CMOS image sensor and a color filter array (CFA) to record one of the three color channels at each pixel location. The primary output of these image sensors is raw RGB. CFA image interpolation, also known as demosaicing, is required to reconstruct a full-color RGB image from raw RGB pixel data [37]. The most common CFA pattern is the Bayer pattern [38], as shown in Figure 4.

Demosaicing methods can be grouped into two groups. The first group of algorithms works on each color channel separately. They include nearest-neighbor replication, bilinear interpolation, and cubic spline interpolation. The second group of algorithms exploits inter-channel correlation. One approach in this group is smooth hue transition, which assumes that the hue does not change abruptly between neighboring pixel locations. In the first step, these algorithms interpolate the luminance (green) channel, which is usually achieved using bilinear interpolation. The chrominance channels (red and blue) are estimated from the bilinearly interpolated “red hue” (red-to-green ratio) and “blue hue” (blue-to-green ratio). Another approach that exploits inter-channel correlation is an edge-directed interpolation. Here, the bilinear interpolation of the green channel is replaced by adaptive interpolation to prevent interpolation across edges [39].

Gunturk et al. [39] presented a new demosaicing algorithm that uses inter-channel correlation in an alternating-projections scheme. The proposed algorithm was compared to bilinear interpolation and six other demosaicing algorithms; it demonstrated an outstanding performance both visually and in terms of mean square error at reasonable computational complexity. Kimmel [40] presented a simplified image formation model, used to reconstruct an algorithm for image reconstruction with a CCD sensor, based on Bayer CFA. The performance of the developed interpolation algorithm was tested on four benchmark images. Bilinear interpolation was used as the reference method to show the visual improvement offered by the proposed scheme. Lukac et al. [41] presented a cost-effective edge-sensing correlation–correction interpolation method for digital still cameras that uses edge-sensing weighted coefficients. To measure the efficiency of this method, a color image was first transformed into a Bayer image. The efficiency of the interpolation methods was measured using the mean square error and the normalized color difference criterion. The method was compared with seven other methods on the twenty color images. On average, they achieved a 20–30% improvement. Bailey et al. [42] implemented a demosaicing algorithm using an Altera Cyclone V FPGA. They recommend integrating the Bayer interpolation within the FPGA image-processing pipeline when the pixels are streamed from the image sensor. In the first stage of their three-stage algorithm, the missing green pixels were first estimated using a high-order interpolation in four directions, with a 7 × 7 interpolation window. The second stage used a simple first-order interpolation and interpolated diagonally to estimate the missing blue and red pixels. The final stage was used to interpolate the red and blue channels both horizontally and vertically. Images with a resolution of 800 × 600 and an 8-bit pixel depth were captured and displayed on a VGA monitor. In the results, they compared the resource requirements of the proposed algorithm with the other five algorithms. The quality of the processed images was estimated visually, and the authors observed significantly better quality, although at the cost of increased resource utilization.

### 2.4. Color to Grayscale Image Conversion

In most image-recognition applications, digital image processing is conducted on grayscale images. Edge detection is one of the most frequently used procedures in digital image processing. Many researchers have proposed an FPGA implementation of some edge detection algorithm, and their first step is usually RGB-to-grayscale conversion. Color edge detection is rare, because its calculation requirements are three times greater than in the case of a grayscale image, and 90% of the edges are approximately the same in grayscale and color images [43]. Converting color images to grayscale is used for various reasons, such as for reproducing monochrome devices. Color-to-grayscale conversions reduce three-dimensional color data into a single dimension. During this process, some loss of information is inevitable. The goal is to preserve as much information as possible and to produce perceptibly plausible grayscale results. Various approaches have been proposed. One simple, widely used, and computationally efficient approach is based on neglecting the chrominance channels (red and blue) and taking the luminance channel (green) as a representation of the original color image [44]. In 2005, Gooch et al. [45] presented the Color2Gray algorithm with the aim of improving what at that time was considered conventional algorithms (CIECAM97 Lum, L*a*b* Lum, XYZ Lum, YCrCb Lum, Photoshop Auto Contrast) for converting RGB images to grayscale. These algorithms, which employ a simple pixel-weighted sum to map a 3D color space to a single dimension, are ineffective at preserving chrominance differences between isoluminant pixels. The Color2Gray algorithm encodes differences from a color image into luminance differences in grayscale. The Color2Gray algorithm does not provide large improvements for scenes with a high dynamic range. However, the method does improve any image that contains large isoluminant regions with a small number of different chrominance values. The algorithm is computationally very intensive. In 2005, using an Athlon 64 3200+ processor, computing images with full neighborhoods required 12.7 s for a 100 × 100-pixel image.

## 3. Methods and Materials

The FPGA image-processing block is the functional core of our device. The device performs the function of sharp grayscale image acquisition for an illuminated eye, using a camera module that integrates a color-image sensor and a motorized lens. The FPGA image-processing block is supported by the IR distance measurement circuit and by the visible light illumination. Both light sources conform to the IEC62471 safety standard. In the following section, we first present the FPGA image-processing block; then, we present the supporting hardware of the eye-image acquisition device, the experimental setup, and the test procedures.

### 3.1. FPGA Image-Processing Block

The FPGA image-processing block performs two very important tasks:

1. Image conversion from 10-bit raw RGB to 8-bit grayscale;
2. Image sharpness value calculation.

Both image-processing blocks run in parallel and in real time on the FPGA. The combined result of both operations is a sharp 8-bit grayscale image from the camera module that is based on a low-cost 10-bit color image sensor.

**Figure 5:**
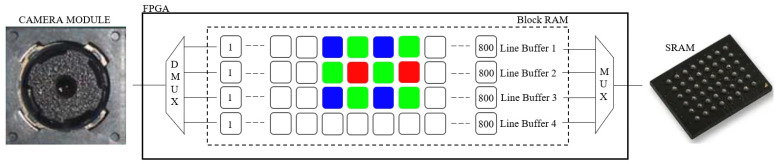
FPGA real-time image processing block.

The pixel-data flow from the camera module to the external SRAM, through the FPGA real-time image-processing block, is shown in Figure 5. See also Figure 4. In this block, four line-buffers (LBs) are implemented as block RAM (BRAM) cells inside the FPGA. Each LB holds an entire SVGA line or 800 pixels. LBs are filled with SVGA lines sequentially, line by line. When the fourth LB is full, the next SVGA line is written in the first LB, etc., until all 600 lines are acquired and processed. Each LB’s cell location can be accessed (write-to LB cell or read-from LB cell) in a single FPGA clock. For example, when writing 10 bits of pixel data in any of the fourth LB cells, 10 bits of pixel data can be simultaneously red from any cell in the first, second, or third LB. This is the basis of our FPGA image-processing block. Each processed pixel is written to the external SRAM for further processing. In our case, this entails displaying the processed image to the SVGA display or sending the processed image to the PC over the UART-USB interface.

**Figure 6:**
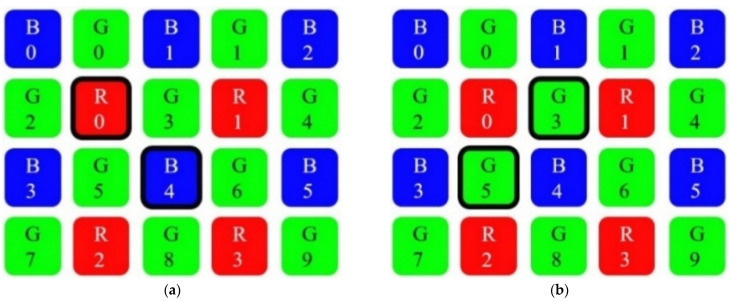
Pixels involved in real-time FPGA image processing: (**a**) edge-directed green color bilinear interpolation; (**b**) image sharpness value calculation from red and blue pixels.

The camera module was set to the raw-RGB image data format. Each acquired pixel had a 10-bit intensity value of either red, green, or blue color. Half of all of the pixels in the image sensor were green. Therefore, the spatial resolution of green pixels was twice that compared to the blue pixels’ or red pixels’ spatial resolution. We interpolated the missing green pixels to obtain an 8-bit green monochrome image that can be approximated to a grayscale image, which we would obtain from an 8-bit monochrome grayscale image sensor. To interpolate the missing green pixels, we used an edge-directed bilinear interpolation. Figure 6a, Equations (1) and (2) show the pixels involved in an edge-directed bilinear interpolation of the missing green pixels.

Green color interpolation was calculated at every red pixel position and at every blue pixel position. The image sharpness value was calculated at every green pixel position as a blue-gradient and red-gradient calculation. By doing this, the two image-processing operations were interleaved on a single image frame. To the best of our knowledge, the described image processing method has not yet been presented in any iris image acquisition application. Figure 6b and Equations (3)–(5) show how the green pixels’ positions are used to calculate the image sharpness of an image frame. For an image frame with 480,000 pixels, there are 240,000 pixel-sharpness calculations. Better focused images have higher image sharpness values.

Interpolating a green pixel at the red-R0 pixel position into a grayscale 8-bit value:

if |G2 − G3| > |G0 − G5|=> interpolated green pixel value = (G0 + G5)/8 
if |G2 − G3| < |G0 − G5| => interpolated green pixel value = (G2 + G3)/8
(1)

Interpolating a green pixel at the blue-B4 pixel position into a grayscale 8-bit value:

if |G5 − G6| > |G3 − G8| => interpolated green pixel value = (G3 + G8)/8 
if |G5 − G6| < |G3 − G8| => interpolated green pixel value = (G5 + G6)/8(2)

Pixel sharpness calculation at the green-G3 pixel position:

pixel_sharpness (k) = |R0 − R1| + |B1 − B4|(3)

Pixel sharpness calculation at the green-G5 pixel position:

pixel_sharpness (k) = |B3 − B4| + |R0 − R2|(4)

Image sharpness = [∑^k^ pixel_sharpness (k)]/64 (5)

### 3.2. Supporting Hardware of the Eye-Image Acquisition Device

**Figure 7:**
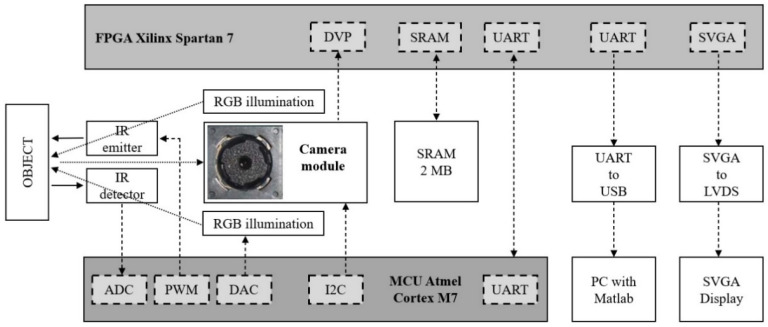
Functional block diagram of the developed eye image acquisition device.

The developed eye-image acquisition device was designed around a camera module. We used a small, low-cost, and easy-to-purchase camera module with an integrated image sensor and motorized lens. Figure 7 shows the functional block diagram of the developed eye-image acquisition device, with the following core components:

- Camera module [46] as the image acquisition device;
- Microcontroller (MCU) [47] as the system controller;
- FPGA [48] with 2 MB external SRAM memory [49] as the image processing core;
- Illumination subsystem for object illumination based on RGB LEDs [50];
- Distance measurement subsystem to measure the distance between the object and the camera module based on IR emitting LEDs [51] and IR detecting photodiodes [52];
- External PC that is connected to the FPGA through the external UART/USB chip interface [53], for off-line MATLAB [54] image processing and testing;
- External 8.4” SVGA display [55] that is connected to the FPGA through the external SVGA/LVDS chip interface [56], for real-time image debugging. The display is used in the prototype device for debug purposes only, and it will not be integrated into the final product.

The camera module integrated a 5 M pixel CMOS color image sensor and a motorized lens to adjust the camera focus. The image size, the image data format, and the focus lens position were adjusted over the I2C bus by the MCU. In our design, the image size was set to SVGA (800 × 600 pixels), and the image data format was set to raw RGB, where each pixel was 10-bit deep. The image frames were captured by the FPGA over the digital video port (DVP).

**Figure 8:**
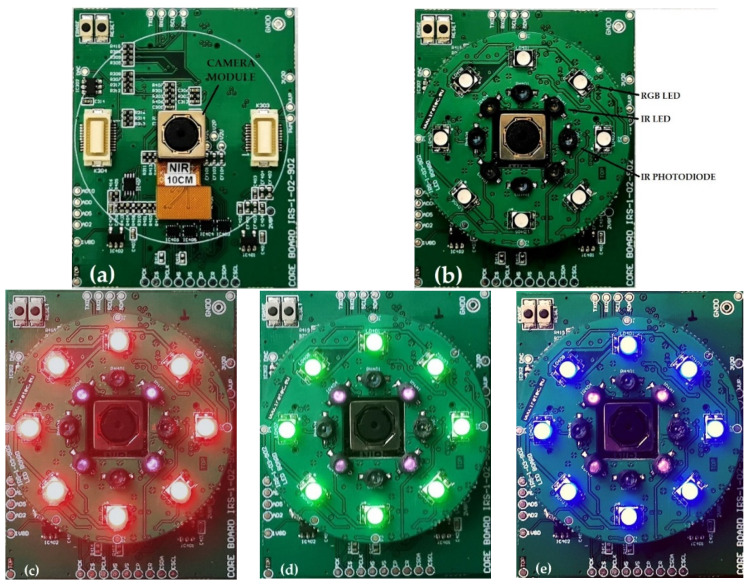
Image acquisition electronics: (**a**) camera module mounted on the MCU PCB; (**b**) IR distance/RGB illumination PCB mounted on the MCU PCB; (**c**) red RGB LED turned on; (**d**) green RGB LED turned on; (**e**) blue RGB LED turned on.

Figure 8a shows the camera module mounted on the CPU board. The SVGA image size was selected to obtain eye images with good enough resolution that would fit the display’s size. However, this also means that our device is not compliant with the ISO/IEC19794-5 standard. Since we were using a local on-chip database, which is not accessible by the external world, this is not an issue.

The image pixel clock was set to 12 MHz, which gives an approximate frame rate of 5 frames per second. At this pixel clock, the frame duration was 195 ms. This frame rate was selected due to the timing requirements of the FPGA real-time image-processing block. The FPGA chip runs at 200 MHz. This provides the processing block with 16 clock cycles to process each incoming pixel in real time.

The image sharpness was adjusted by adjusting the position of the integrated motorized lens. To speed up the focusing process, the camera’s lens position was first set roughly, according to the measured distance between the camera module and the object. This was achieved by the IR distance measurement circuit, where the main components are four IR LEDs and four IR photodiodes. The emitted and reflected IR light was detected by the IR photodiodes when an object was placed in front of the camera module. The distance measurement circuit, arranged around the camera module, is shown in Figure 8b.

Since we used a camera module with an integrated CFA-based image sensor, which is sensitive to red, blue, and green light wavelengths, a three-color RGB LED illumination was used, with peak wavelengths at 634 nm, 522 nm and 465 nm for the red, green, and blue illumination channels, respectively. As can be seen in Figure 8b, there were eight RGB LEDs arranged symmetrically around the camera module. The illumination intensity for each illumination channel can be adjusted independently by the MCU, according to the measured distance from the object, while considering the eye-safety illumination limits. In Figure 8c–e, the red, green, and blue LEDs are turned on. The advantage of using visible illumination over IR illumination is that it can be used for eye liveness detection, since the eye pupil changes in size when the visible light illumination level is changed. The eye image was acquired at a short distance of 7–13 cm. Therefore, the visible illumination should ensure capturing enough iris structure detail for the iris recognition process.

**Figure 9:**
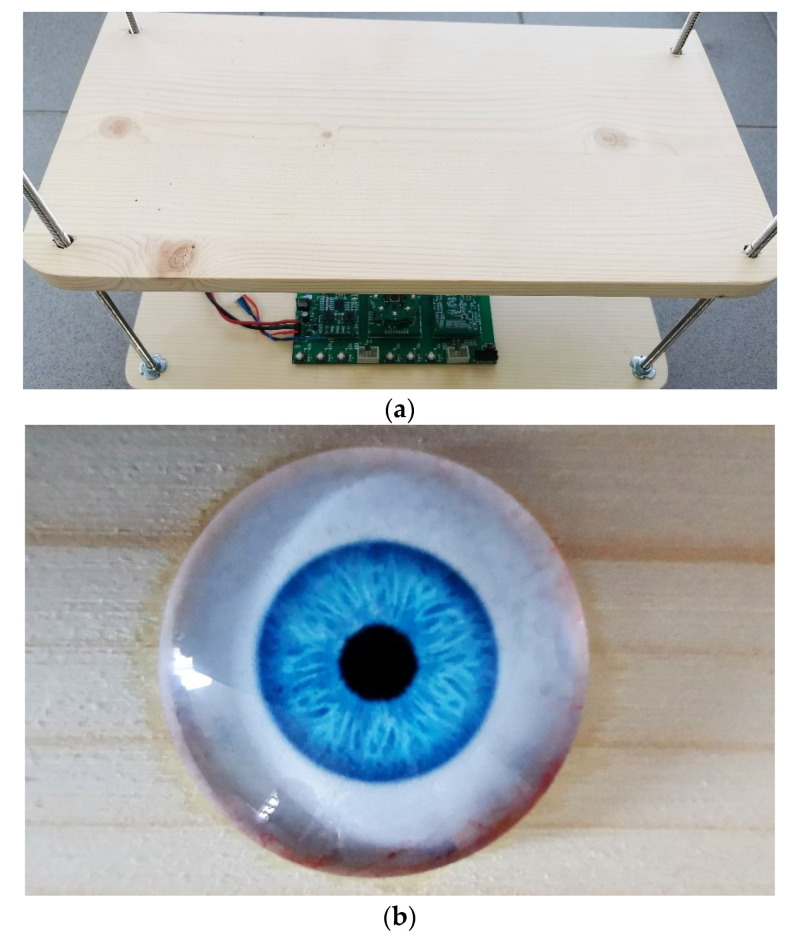
Eye image acquisition experimental setup: (**a**) developed prototype electronics under the toy eye; (**b**) toy eye glued on the top board.

To acquire a sharp 8-bit grayscale image using our eye-image acquisition device, we used a simple experimental setup, as shown in Figure 9a. The setup was composed of two wooden boards, four metal threaded sticks, and four screw nuts under the top wooden board. The distance between the camera module and the top boards was adjusted manually by adjusting the position of the nuts. Instead of acquiring real-eye images, a plastic artificial toy eye with a diameter of 22 mm [57], as shown in Figure 8b, was used. At this stage of the project, we did not use real human eyes, since we were interested in the core functionality and the concept of the proposed eye-image acquisition device.

### 3.3. Experimental Setup and Test Procedures

In the first step, the experimental setup was used to calibrate the IR distance measurement circuit. In the second step, the sharpness function as a function of distance was measured. In the last step, the experimental setup was used for testing the proposed eye-image acquisition scheme. Using the calibrated IR distance measurement circuit, the focus lens position is first set roughly. Then, the fine-tuning process of the lens position adjustment may start. At this point, the RGB illumination begins and the passive focusing algorithm is executed. Here, image sharpness values are calculated from sequentially acquired frames. Fine adjustment of the camera’s lens position is conducted, based on current and previous image sharpness values. The camera’s lens position is adjusted by the MCU over the I2C bus in the range from 0 to 1023 digits. The image-focusing process is finished when a maximum of six consecutive images are acquired. Therefore, the whole image-focusing process never takes more than 1200 ms. At this point, we acquired an image with a maximum image sharpness value. The RGB illumination level was set according to the measured distance from the eye.

**Figure 10:**
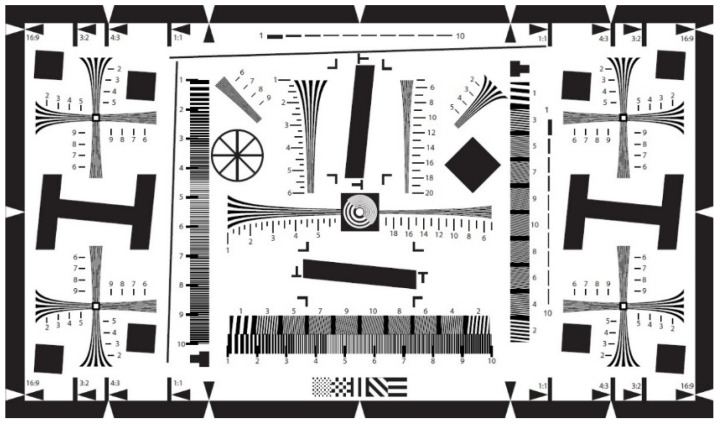
ISO-12233 chart for image sharpness evaluation [58].

To measure the image sharpness function as a function of distance from the camera, the ISO12233 chart was used as the focusing target image. A PDF image file version of this chart is shown in Figure 10. The chart was obtained from [58], printed, and fixed on the bottom side of the top wooden board of the mechanical setup.

It is important that the RGB illumination circuit and the IR distance measurement circuit comply with the IEC62471 standard. To calculate the total hazard exposure level of a single RGB LED at a 10-mm distance and the hazard exposure level of a single IR LED at a 10-mm distance, the radiant power Φ [W] of a red, green, blue, and IR illumination was measured first. The standard IEC62471 [59] and the power-meter setup from Gentec [60,61] were used for this purpose. Here, worst-case illumination scenarios were assumed, as the eye would be centered 10 mm above an LED and illuminated for 5 s. Equations (6) and (7) show the calculation of the retinal blue light hazard exposure limit (EB × t, EB) of a small light source for the spectral region from 300 to 700 nm. Equations (8) and (9) show the calculation of the infrared radiation hazard exposure limit (EIR) for the eye for the IR spectral region from 780 to 3000 nm. E(λ) is the spectral irradiance (W × m^2^ × nm^−1^), defined as the quotient of the radiant power dΦ(λ) in a wavelength interval dλ, incident on an element of a surface, by the area dA of that element, and by the wavelength interval dλ. λ is the wavelength (nm), Δt is the exposure time (s), Δλ is the bandwidth (nm), and B(λ) is the blue-light hazard weighting function (constant).

EB × t = ∑λ∑t E(λ) × B(λ) × Δt × Δλ ≤ 100 J/m^2^   valid for t ≤ 100 s (6)

EB = ∑λ E(λ) × B(λ) × Δλ ≤ 1 W/m^2^   valid for t > 100 s (7)

EIR = ∑^λ^ E(λ) × Δλ ≤ 18.000 × t^−0.75^ W/m^2^    valid for t ≤ 1.000 s (8)

EIR = ∑^λ^ E(λ) × Δλ ≤ 100 W/m^2^    valid for t > 1.000 s (9)

## 4. Results and Discussion

**Figure 11:**
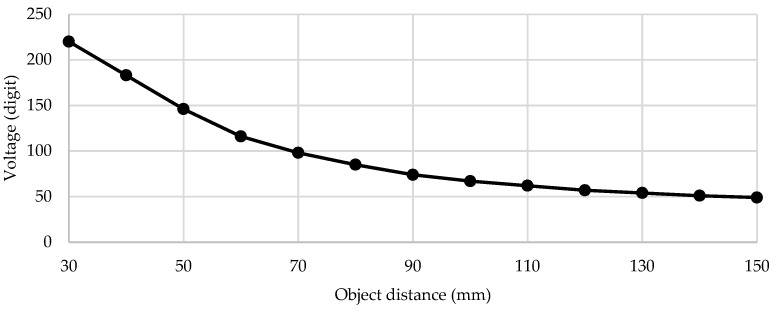
Measured voltage/distance characteristics of the distance measurement subsystem.

**Figure 12:**
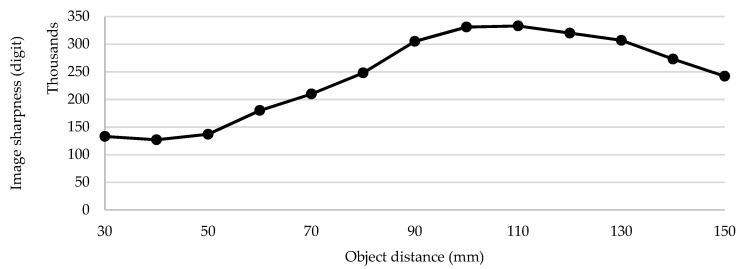
Measured image sharpness as a function of distance from the object.

**Figure 13:**
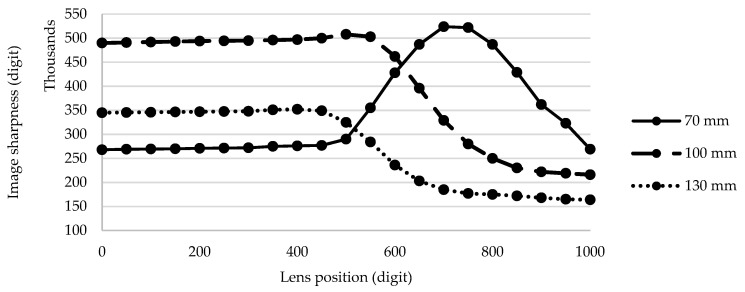
Measured image sharpness as a function of lens position at 3 fixed distances from the object: 70 mm, 100 mm, and 130 mm.

In this section, we present the results of the tests that were obtained using the experimental setup presented in Section 3. Figure 11 shows the measured voltage/distance characteristic. The voltage of the IR detection circuit was sampled by the MCU’s 10-bit ADC at a 3.3 V reference voltage. A look-up table and linear interpolation between calibration points were used in the code for voltage-to-distance conversion. Figure 12 shows the measured image sharpness function of the developed imaging system in terms of image sharpness versus object distance. As specified by the manufacturer, the maximum value of the sharpness function was at a 100-mm distance. Figure 13 shows the measured image sharpness as a function of the lens position at three fixed distances from the object: 70 mm, 100 mm and 130 mm. As expected, the best performance of the image-sharpness circuit was measured at a 100-mm distance. This is the optimal standoff acquisition distance of the system.

The developed eye-image acquisition device was tested at a distance range of 7–13 cm from the target. The measured span of the distance measurement subsystem in this range was approximately 50 digits. The achieved distance measurement accuracy was good enough to perform active focusing with this application.

**Figure 14:**
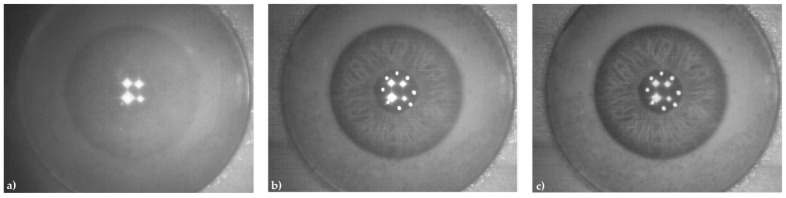
Acquired 8 bit grayscale images of the artificial eye at distance 100 mm, at a focus lens position of 590 digits, and under different illumination conditions: (**a**) no illumination (only the infrared distance measurement circuit is active), image sharpness = 136,000; (**b**) low RGB illumination level, image sharpness = 195,000; (**c**) high RGB illumination level, image sharpness = 229,000.

To estimate the overall performance of the proposed eye-image acquisition device, the subjective visual approach and the image sharpness value metric were used. Figure 14 shows three acquired images under different illumination conditions in the best focus position. Image sharpness improved significantly with higher illumination levels. In ten attempts, the average sharp image acquisition time was 1 s, with acquisition times ranging from 800 to 1200 ms. The diameter of the acquired iris was approximately 400 pixels, which is double compared to the standardized 200 pixels. This will be an advantage when the whole iris recognition system is implemented. The undesired illumination artifacts were well concentrated inside the pupil area.

Table 2 shows the calculation of the total retinal blue hazard exposure of a single RGB LED. Table 3 shows the calculation of the infrared radiation hazard exposure of the eye to a single IR LED. Both calculated hazard exposure levels were below their hazard exposure level limits. The retinal blue hazard exposure level limit, EB × t, is 100 J/m^2^. The calculated infrared radiation hazard exposure level limit, EIR, was 5883 W/m^2^.

## 5. Conclusions and Future Work

The purpose of this work was to verify if a compact access-control device based on iris recognition technology, intended for easy integration into smart-city doors, could be built at a reasonable cost. In every iris recognition system, the image acquisition subsystem represents the most complex and expensive part. Therefore, the project was divided into two stages. In this work, we presented the results of the first stage, where the goal was to develop and test just the eye-image acquisition part of the system.

The results showed a good performance by the developed device. We managed to acquire a sharp image of an artificial toy eye sized 22 mm in diameter from an approximate distance of 10 cm, achieving 400 pixels over the iris diameter, with an average acquisition time of 1 s, and with illumination levels below hazard exposure levels.

The main drawback of this work was the limitations induced by the usage of an artificial toy eye and, consequently, the significantly constrained iris image acquisition conditions. We were unable to measure the performance of the complete iris recognition system, which can be achieved by using real eye images, less unconstrained eye image acquisition, and implementing all iris recognition phases.

Nevertheless, we obtained enough positive data to continue our work, which will be focused on the development of a complete iris recognition system for smart-city access control applications. In the second stage, the device will be further miniaturized and other iris recognition phases (iris segmentation, iris coding, code-matching) will be added as software upgrades. The optimal visible illumination levels for different iris colors as a function of the distance from the camera will also be studied and implemented. The performance of the whole iris recognition system will be measured in terms of its access control accuracy metrics, such as the false accept rate (FAA) and false reject rate (FRR).

## Figures and Tables

**Table 1:** Typical technical characteristics of products from smart-city access control applications, based on iris recognition.

Company/Product	Size W × H × D (mm)	Capture Range (cm)	Image Sensor Resolution	Access Time (seconds)	Illumination Wavelength (nm)	Number of Users	Compliance Standard	Mount Type
IRISID/iCAM7	178 × 211 × 64	28–38	640 × 480	Data not available	Data not available	10,000	ISO19794-6 IEC62471	Wall-mount
GRANDING/ IR7pro	193 × 155 × 43	17.5–40	2304 × 1536	2.3	Data not available	1000	Data not available	Wall-mount
IRITECH/ MO2121 + camera	36 × 40 × 7 48 × 18 × 8	13–14	640 × 480	0.5	700–900	500	ISO19794-6 IEC62471	OEM module
THOMAS/ TA-EF-45	166 × 166 × 43	35–45	640 × 480	1.0	700–900	10,000	ISO19794-6 IEC62471	Wall-mount
Proposed concept design	40 × 40 × 40	7–13	800 × 600	<2.0	465–634	30	IEC62471	Door-mount

**Table 2:** Total retinal blue hazard exposure calculation of a single RGB LED.

LED	Wavelength ^1^ λ (nm)	LED Current ^2^ IL (mA)	Radiant Power ^3^ Φ (mW)	Area ^4^ A (m^2^)	Weight B(λ) (Constant)	Exposure Time ^5^ Δt (s)	Bandwidth ^6^ Δλ (nm)	Hazard Exposure Level (J/m^2^)
1× Red	634	20	0.6	9.42 × 10^−4^	0.001	5	30	0.153
1× Green	522	10	0.91	9.42 × 10^−4^	0.0363	5	65	11.40
1× Blue	465	4	0.55	9.42 × 10^−4^	0.7	5	40	81.74
**Total**								93.29

^1^ Peak wavelength data were used (see: ASMB-MTB0-0A3A2 [50], Table 2). ^2^ Maximum LED currents of the current control circuit. ^3^ Measured by [58,60] just above the LED package. ^4^ Radius of (*r* = 10 mm × tg (60°) = 17.32 mm) of the circular area at a distance of 10 mm from the LED package was assumed at a viewing angle θ/2 = 60°. ^5^ Limited to 5 s by the software. ^6^ Estimated from the normalized intensity characteristics (see: ASMB-MTB0-0A3A2 [50], page 5, Figure 1, bandwidth red at 0.5 normalized intensity).

**Table 3:** Infrared radiation hazard exposure for the eye of a single IR LED.

LED	Wavelength ^1^ λ (nm)	LED Current ^2^ IL (mA)	Radiant Power ^3^ Φ (mW)	Area ^4^ A (m^2^)	Exposure Time ^5^ Δt (s)	Bandwidth ^6^ Δλ (nm)	Hazard Exposure Level (W/m^2^)
1× Infrared	940	100	0.425	1.27 × 10^−4^	5	40	133.9

^1^ Peak wavelength data were used (see: VSMB2020X01 [51], page2, peak wavelength). ^2^ The LED driving current is set by hardware with 50% duty-cycle. ^3^ Measured with [58,60] just above the LED package. ^4^ Radius of (*r* = 10 mm × tg (12°) = 6.36 mm) of the circular area distant 10 mm from the LED package was assumed at a viewing angle θ/2 = 12°. ^5^ Limited to 5 s by the software. ^6^ Estimated from the relative radiant power characteristics (see: VSMB2020X01 [51], Page 3, Figure 5, bandwidth red at 50% of relative radiant power).

## Data Availability

Data are available upon request.
